# Relationship of coping ways and anxiety with Pregnancy Specific-stress

**DOI:** 10.12669/pjms.326.10892

**Published:** 2016

**Authors:** Mahbobeh Faramarzi, Fatemeh Nasiri Amiri, Razieh Rezaee

**Affiliations:** 1 Mahbobeh Faramarzi, Associate Prof. of Social Determinants of Health Research Center, Babol University of Medical Science, Babol, Iran; 2 Fatemeh Nasiri Amiri, Assistant Professor of Midwifery, Fatemeh Zahra Fertility & Infertility Research Health Center, Babol University of Medical Science, Babol, Iran; 3 Razieh Rezaee, Student Research Committee, Babol University of Medical Science, Babol, Iran

**Keywords:** Hassles, Uplifts, Ways of coping, general anxiety, Pregnancy Specific-stress

## Abstract

**Objectives:**

To explore whether coping strategies and general anxiety are associated with pregnancy-specific stress (PSS) and how much of variance of PSS is explained with these variables.

**Methods:**

A cross sectional study was conducted at two teaching hospitals between November 2013 and December 2015. Total 190 pregnant women (60 women at 6-13-weeks of gestation, 60 at 13-26 weeks, and 70 at 27-40 weeks of gestation) completed the study. The participants completed three questionnaires including; Pregnancy experience scale (PES-41), Ways of Coping Questionnaire (WCQ), and State-Trait anxiety inventory (SATI). Pearson coefficients and analysis of regression was done to assess the correlations between variables.

**Results:**

Pregnant women who experienced higher mean level of pregnancy specific-stress had significantly higher mean level of occult anxiety, overt anxiety, and total anxiety than women who did not experience PSS. Although there was a positive and significant relationship between intensity of hassles and uplifts and ways of coping, the correlation between PSS and ways of coping was not significant. The results of analysis regression showed that general anxiety during pregnancy predicted 25% of the variance of PSS (F=4.480, β=0.159). Also, ways of coping predicted 38% of the variance in pregnancy Hassles (F=7.033, β=0.194).

**Conclusion:**

The ways of coping predicted the variance of pregnancy hassles, but does not evaluate pregnancy specific-stress. To think about PSS in terms of general anxiety may help to clarify past findings and to guide future research and interventions.

## INTRODUCTION

Pregnancy-specific stress (PSS) is defined as worries, concerns and fears of mother related to pregnancy.^1^ PSS includes labor and childbirth, physical symptoms parenting, and relationships with others. Pregnancy is a time with much stress, hassles and uplifts experiences.^2^,^3^ Hassles in daily life focuses on the irritating, frustrating, “unpleasant,” and “aversive,” events. Evidence support assessing uplifts (pleasant events) along with hassles are specific to pregnancy.^4^

During pregnancy, hassles and uplifts result from stress increase and decrease. PSS indicate more hassles than uplifts.^4^

A large number of studies have demonstrated that general pregnancy stress is associated with a variety of negative consequence for both mother and fetus, including premature birth,^5^ higher risk of developing hypertension and preeclampsia,^6^ and increased risk of miscarriage or stillbirth.^7^ Lobel et al. reported that pregnancy-specific stress may be more related to birth outcomes than general stress.^8^

Coping strategies have been defined as any attempt to manage conditions that are perceived as stressors.^9^ Coping efforts may influence birth outcomes by reducing or preventing negative emotional, behavioral, cognitive and physiological responses to stressors.^10^ Adapting coping strategies such as active and problem-focused coping strategies resolve the stressor and thereby protect against adverse birth outcomes, but maladaptive forms of coping (such as avoidance-to escape from feeling of distress related to the stressor) are passive and less effective.^2^ Maladaptive coping strategies are associated with negative emotional outcomes.^11^ Evidence support that coping strategies are as moderators of psychological distress such as depressive symptoms.^12^

A recent systematic review coping during pregnancy recommended that it is better to use of pregnancy-specific and daily process.^13^ The current study addresses the existing gap in the pregnancy stress literature based on testing the relationship of coping strategies and general anxiety with pregnancy specific stress. To the authors’ knowledge, this is the first study to examine the association between three variables. The aims of the study were:

1. To explore if coping strategies are associated with pregnancy-specific stress.
2. To explore if general anxiety is associated with pregnancy-specific stress.
3. To explore how much of variance of pregnancy-specific stress is explained with coping strategies and general anxiety.

## METHODS

This cross-sectional study was conducted between November 2013 and December 2015 in

190 pregnant women of two teaching hospitals of Babol University of Medical Sciences. We included all women referred to the clinics at 6-13 weeks, 13-26 weeks, and 27-40 weeks. Eventually 210 women participated in the survey, 190 completed it (60 women at 6-13-weeks, 60 at 13-26 weeks, and 70 at 27-40 weeks).

Only women who had education of at least 5 years were included. All subjects gave their informed consent prior to inclusion in the study which was approved by the Medical Education Ethics Committee of Babol University of Medical Sciences.

Two midwives recorded the demographic and pregnancy information. Also, the midwives distributed the study questionnaires to the respondents during prenatal visit. They gave a brief explanation regarding the purpose of the study, reminded the subjects of their rights not to answer any question if they wished to, and how to fill in the questionnaires. The participants completed three questionnaires including; Pregnancy experience scale (PES-41), Ways of Coping Questionnaire (WCQ), and State-Trait anxiety inventory (SATI).

PES-41 was developed by Dipietro et al. to measure hassles and uplifts to asses’ specific- pregnancy. The questionnaire consisted of a list of 41-items each rated on a 4-point Likert scale for both uplifts and hassles. It ranged from 0 (not at all) to 3 (a great deal). Subjects are directed to indicate whether each item is considered as either uplifts or hassles. A composite ratio score relating hassles to uplifts for intensity scale scores was computed to assess the balance of positive versus negative. Values lower than one indicates more uplifts than hassles. Also, scores greater than one indicate more hassles than uplifts.^4^ A forward–backward procedure was applied to translate the English version of the PES-41 into Persian (the Iranian language). The validity of the Iranian language version of the PES-41 was found to be high (0.68 Cronbach’s alpha).

WCQ is a scale that identifies thoughts and actions that people use to cope with the stressful events counters of everyday living. This instrument contains 66 items and 8 subscales. The subscales consist of seeking social supports (talking to others about the problem), distancing (making light of the stressful events), planful problem-solving (seeking to solve the problem), escape/avoidance (avoiding people), confrontive coping (directly challenging the problem), self-control (trying to keep emotion from interfering with activities), accepting responsibility (believing one is responsible for stressful events), and positive reappraisal (reevaluating the stressful events to find unexpected benefits). In this study, we used a valid Persian version of the WCQ.^14^

STAI is a self-reporting questionnaire for anxiety symptoms. It consists of 20 statements: “state anxiety” (set relates to immediate situation) and “trait anxiety” (set is intended to reflect underlying long-term anxiety). The responders are required to select a single response for each statement: almost never (1), sometimes (2), often (3), and all the time. Range of scores for each subscale is 20 and 80, the higher score indicating greater anxiety.^15^ The valid Persian STAI was used in Iran.^16^

Data were analyzed using SPSS version 20. We used as frequencies and percentages and quantitative as mean and standard deviation (SD) to describe the characteristics of population study. T student test was used to compare differences in women with pregnancy specific-stress and without PSS. Pearson coefficients and analysis of regression were used to assess the correlations between variables. P < 0.05 was considered for level of significance.

## RESULTS

The characteristics of the population study are shown in Table-I. The mean age of the participants was 24.17 ± 5.30 years (range 15–41 years). 5.8% of the subjects were employees. 18.3% of the population had educating of more than 12 years.

**Table-I:** Demographic characteristics of the study population.

Variables	First trimester N=60	Second trimester N=60	Third trimester N=70
Age (Mean, SD)	24.27± 5.04	23.6±6.1	24.4±4.9
Gestational age	8.0± 2.1	18.4± 8.3	31.5± 9.1
*Job (N, %)*			
House worker	55 (91.7)	48 (80)	66 (94.3)
Employee	5 (8.3)	12 (20)	4 (5.7)
*Education (N,%)*			
>12 years	47 (79.3)	52 (89)	58 (82.8)
≤12 years	13 (21.7)	8 (11)	12 (17.2)
*Parity (N,%)*			
0-1	28 (47.4)	40 (66)	59 (84.2)
≤2	31 (52.6)	20 (24)	11 (15.8)

The comparison of means and standard deviations of ways of coping and anxiety in two groups of with and without pregnancy specific-stress is depicted in Table-II. Two groups did not have a meaningful difference in the mean of total ways of coping, all 8 types of coping, and two subscales of the (emotional coping and problem solving coping) of the WCQ. Pregnant women who experienced higher mean level of pregnancy specific-stress had significantly higher mean level of occult anxiety, overt anxiety, and total anxiety than women who did not experience pregnancy specific-stress (*p* < 0.05).

**Table-II:** Comparison of means ways of coping and anxiety in two groups of with and without pregnancy specific-stress.

Variables	Without pregnancy specific-stress (N=130)		With pregnancy specific-stress (N=42)		t	P value

	Mean	SD	Mean	SD		
Confrontive coping	7.67	4.63	6.95	3.22	1.13	0.26
Distancing	8.55	4.57	8.26	3.21	0.45	0.64
Self-controlling	10.50	5.07	10.11	4.44	0.43	0.66
Seeking social supports	9.72	4.61	8.78	3.99	1.18	0.23
Accepting responsibility	6.20	3.29	6.40	2.76	-0.36	-0.20
Escape/Avoidance	9.87	6.16	6.40	2.76	-0.13	-0.14
Planful problem-solving	8.38	4.89	8.26	3.49	0.17	0.85
Positive reappraisal	11.76	5.59	11.38	4.28	0.46	0.64
Emotional copings	36.60	18.97	35.35	14.65	0.44	0.65
Problem- Solving copings	36.06	17.26	34.83	12.94	0.49	0.62
Total Ways of Copings	72.67	35.62	70.19	26.37	0.48	0.62
Anxiety Occult	43.19	9.11	47.39	10.73	-2.50	0.01
Anxiety Overt	43.04	9.56	45.80	8.87	-1.65	0.09
Total general anxiety	86.24	17.91	93.42	18.45	-2.24	0.02

The bivariate correlations among pregnancy specific-stress, ways of coping, and anxiety is shown in Table-III. It can be concluded that although there was a positive and significant relationship between intensity of hassles and uplifts and ways of coping, the correlation between PSS and ways of coping was not significant. Moreover, there was a positive and significant relationship between both overt anxiety and occult anxiety and all of the ways of coping and PSS, except occult anxiety and PSS.

**Table-III:** Correlation matrix of the variables.

	1	2	3	4	5	6	7	8	9	10	11	12	13	14	15
1-WCQ1															
2- WCQ2	0.77**														
3- WCQ2	0.79**	0.86**													
4- WCQ2	0.77**	0.74**	0.78**												
5- WCQ2	0.76**	0.78**	0.83**	0.78**											
6- WCQ2	0.83**	0.78**	0.86**`	0.76**	0.81**										
7- WCQ2	0.77**	0.80**	0.84**	0.80**	0.80**	0.83**									
8- WCQ2	0.76**	0.80**	0.85**	0.82**	0.80**	0.78**	0.85**								
9- Problem- Solving copings	0.82**	0.84**	0.89**	0.92**	0.90**	0.85**	0.93**	0.95**							
10- Emotional copings	0.91**	0.90**	0.94**	0.82**	0.86**	0.94**	087**	0.86**	0.92**						
11-Total WOC	0.88**	0.89**	0.93**	0.88**	0.89**	0.92**	0.92**	0.92**	0.97**	0.98**					
12-Intensity down	0.15*	0.15*	0.15*	0.12	0.27**	0.20**	0.17*	0.19**	0.20**	0.18**	0.19**				
13-Intensity up	0.22**	0.13	0.17*	0.21**	0.28**	0.19**	0.11	0.22**	0.21**	0.19**	0.21**	0..43**			
14-Pregnancy stress	0.05	0.09	0.09	0.01	0.15	0.11	0.11	0.08	0.09	0.10	0.09	0.81p**	-0.13		
15-Anxiety overt	0.40**	0.30**	0.39**	0.24**	0.40**	0.51**	0.38**	0.31**	0.36**	0.44**	0.41**	0.19**	0.04	0.15*	
16-Anxiety Occult	0.32**	0.29**	0.35**	0.20**	0.36**	0.47**	0.35**	0.24**	0.30**	0.39**	0.36**	0.13**	-0.02	0.14	0.81**

** P<0.01,

* P<0.05, WCQ1; Confrontive coping, WCQ2; Distancing, WCQ3; Self-controlling, WCQ4; Seeking social supports, WCQ5; Accepting responsibility, WCQ6; Escape/Avoidance, WCQ7; Planful problem-solving; WCQ8; Positive reappraisal.

Analysis regression (Model 1) was performed to examine the relationship of ways of coping and pregnancy hassles. The analysis of variance (ANOVA) results from the overall regression equation were statistically significant, F (1, 179) = 7.033, *p* < 0.001. In Table-IV, the model summary from the regression analysis shows that ways of coping predicted 38% of the variance in pregnancy hassles (β=0.194). Also, the results of analysis regression in Table-IV (Model 2) show that general anxiety during pregnancy predicted 25% of the variance in pregnancy specific-stress (F=4.480, β=0.159).

**Table-IV:** Results of analysis regression for dependent variables.

*Model summary (1)*	R		R Square	Adjusted R Square	Std. Error	
	0.194		0.38	0.032	0.72381	
ANOVA Model	Sum of Squares		df	Mean Squares	F	Significant
Regression	3.685		1	3.685	7.033	0.009
Residual	93.779		179	0.524		
Total	97.464		180			
Coefficients	Unstandardized		Standardized	t	Significant	
	B	SD	B			
Constant	1.308	0.124	0.194	2.652	0.009	
WOC total	0.004	0.002				
Model summary (2)	R		R Square	Adjusted R Square	Std. Error	
	0.159		0.025	0.020	0.33893	
ANOVA Model	Sum of Squares		df	Mean Squares	F	Significant
Regression	0.515		1	0.515	4.480	0.036
Residual	19.758		172	0.115		
Total	20.273		173			
Coefficients	Unstandardized		Standardized	t	Significant	
	B	SD	B			
Constant	0.579	0.127		4.556	0.000	
Total general anxiety	0.003	0.001	-0.159	2.117	0.036	

ANOVA Model (1): Way of coping (dependent variable) and pregnancy Hassles (independent variable), ANOVA Model (2): General anxiety (dependent variable) and pregnancy specific-stress (independent variable)

## DISCUSION

Our results showed that although there was a positive and significant relationship between ways of coping and intensity of hassles, the correlation between ways of coping and pregnancy specific-stress was not significant. Some studies have reported contrary to our findings. In one study coping strategies have been shown to mediate the relationship between stress and perinatal mental health.^17^ In another study reported that the use of some coping styles were associated with a significantly reduced occurrence of pregnancy complications.^18^ Some studies are consistent with our results. Wells et al. found that there were no interactions between coping strategies and predicting depression, and no evidence that any of the coping strategies buffered the effect of the stress.^19^ Pakenham et al. found only limited evidence of a stress moderating effect of coping.^20^ The lack of significant interactions between stress and coping in our study and similar studies does not support the hypothesis that coping modifies or buffers the effect of pregnancy specific-stress. In explaining the difference between our results and some studies it can be said that a number of reasons may account for no relationship between coping ways and pregnancy specific-stress. First, situational and interpersonal factors including available resources, competing demands, and the perceived controllability of a situation influence how an individual copes with stress. Second, pregnant women use numerous coping strategies that are related to maternal characteristics, social situations, and emotional reactions. Third, coping is responsive to changing demands across pregnancy and reflective of women’s characteristics, perceptions, and social situations.

This research found that general anxiety during pregnancy is related to PSS and it predicted 25% of the variance in pregnancy specific-stress. Some studies are consistent with our results.^21^ Rallis et al. reported that increased stress scores during mid- pregnancy predicted higher anxiety scores in late pregnancy.^21^ Da Costa et al. assessed the factors associated with elevated hassles, state anxiety and pregnancy-specific stress. 161 subjects completed the Hassles Scale, the pregnancy-specific stress questionnaire, and the state-anxiety scale monthly, beginning in the third month of pregnancy. They found that hassles were stable throughout the pregnancy. Pregnant women reported significantly higher pregnancy-specific stress in the first and third trimester of pregnancy. Poorer marital adjustment predicted higher Hassles during pregnancy and higher pregnancy-specific stress and general anxiety in the third trimester.^22^ Some studies support that there is relationship between psychosocial factors and perceived stress during pregnancy period.^23^ One study has reported that anxiety symptoms during pregnancy contribute to adverse obstetric, fetal and neonatal outcome.^24^ Also, reducing stress during pregnancy is associated with improvement of pregnancy complications.^25^

### Limitations

This study had limitations which should be addressed in future work. Our sample comprised of women that were referred to teaching hospitals, and was predominantly from low to middle class. Also, the processes we have identified may differ for higher-risk samples; especially those who have significant mental health problems, or are experiencing difficult pregnancies. Further research should include women with different socioeconomic status and high risk samples. Our research was a cross-sectional study. Future research would benefit from longitudinal designed through pregnancy and into the postnatal period to assess whether pregnancy specific -stress really does mediate with coping strategies or not. Such research could also explore other possible paths to pregnancy specific-stress, including other characteristics of the women. The novelty of the present study is that it is the first study that has indicated a relationship between pregnancy-specific stress, coping ways, and general anxiety.

## CONCLUSION

Pregnancy specific-stress is not mediated by coping strategies. Thinking about pregnancy specific-stress in term of general anxiety may help to clarify past findings and to guide future research and interventions.

## References

[1] Huizink AC, de Medina PGR, Mulder EJ, Visser GH, Buitelaar JK (2002). Psychological measures of prenatal stress as predictors of infant temperament. J Am Acad Child Adolesc Psychiatry.

[2] Yali AM, Lobel M (1999). Coping and distress in pregnancy: an investigation of medically high risk women. J Psychosomatic Obstetr Gynecol.

[3] Rezaee R, Framarzi M (2014). Predictors of mental health during pregnancy. Iranian J Nurs Midwifery Res.

[4] DiPietro JA, Ghera MM, Costigan K, Hawkins M (2004). Measuring the ups and downs of pregnancy stress. J Psychosomatic Obstetr Gynecol.

[5] Amiri FN, Mohamadpour R, Salmalian H, Ahmadi A (2010). The association between prenatal anxiety and spontaneous preterm birth and low birth weight. Iranian Red Crescent Med J.

[6] Vollebregt KC, Van Der Wal MF, Wolf H, Vrijkotte TG, Boer K, Bonsel GJ (2008). Is psychosocial stress in first ongoing pregnancies associated with pre-eclampsia and gestational hypertension?. Int J Obstetr Gynaecol.

[7] Wisborg K, Barklin A, Hedegaard M, Henriksen TB (2008). Psychological stress during pregnancy and stillbirth: prospective study. Int J Obstetr Gynaecol.

[8] Lobel M, Cannella DL, Graham JE, DeVincent C, Schneider J, Meyer BA (2008). Pregnancy-specific stress, prenatal health behaviors, and birth outcomes. Health Psychology.

[9] Lazarus RS, Folkman S (1984). Stress, appraisal, and coping.

[10] Dole N, Savitz DA, Siega-Riz AM, Hertz-Picciotto I, McMahon MJ, Buekens P (2004). Psychosocial factors and preterm birth among African American and White women in central North Carolina. Am J Public Health.

[11] Yali AM, Lobel M (2002). Stress-resistance resources and coping in pregnancy. Anxiety Stress Coping.

[12] Wei M, Ku T-Y, Russell DW, Mallinckrodt B, Liao KY-H (2008). Moderating effects of three coping strategies and self-esteem on perceived discrimination and depressive symptoms: A minority stress model for Asian international students. J Counsel Psychol.

[13] Guardino CM, Schetter CD (2014). Coping during pregnancy: a systematic review and recommendations. Health Psychol Rev.

[14] Vahedi H (2000). Investigating of being practical, validity, reliability and assessment of coping styles test among adolescence in high school of Tehran. Unpublished master’s thesis.

[15] Spielberger CD (1983). Manual for the State-Trait Anxiety Inventory STAI (form Y) (“self-evaluation questionnaire”).

[16] Panahi-Shahri M (2002). The primary study in validity, reliability and norms of the state-trait anxiety inventory (STAI). MS. Thesis.

[17] Oni O, Harville EW, Xiong X, Buekens P (2011). Impact of coping styles on post-traumatic stress disorder and depressive symptoms among pregnant women exposed to Hurricane Katrina. Am J Disaster Med.

[18] Oni O, Harville E, Xiong X, Buekens P (2015). Relationships among stress coping styles and pregnancy complications among women exposed to Hurricane Katrina. J Obstetr Gynecol Neonatal Nurs.

[19] Wells JD, Hobfoll SE, Lavin J (1997). Resource loss, resource gain, and communal coping during pregnancy among women with multiple roles. Psychology Women Quarterly.

[20] Pakenham KI, Smith A, Rattan SL (2007). Application of a stress and coping model to antenatal depressive symptomatology. Psychology Health Med.

[21] Rallis S, Skouteris H, McCabe M, Milgrom J (2014). A prospective examination of depression, anxiety and stress throughout pregnancy. Women Birth.

[22] Da Costa D, Larouche J, Dritsa M, Brender W (1999). Variations in stress levels over the course of pregnancy: factors associated with elevated hassles, state anxiety and pregnancy-specific stress. J Psychosomatic Res.

[23] Faramarzi M PH (2015). The Role of Social Support in Prediction of Stress during Pregnancy. J Babol Uni Med Sci.

[24] Alder J, Fink N, Bitzer J, Hösli I, Holzgreve W (2007). Depression and anxiety during pregnancy: a risk factor for obstetric, fetal and neonatal outcome? A critical review of the literature. J Matern Fetal Neonatal Med.

[25] Faramarzi M, Yazdani S, Barat S (2015). A RCT of psychotherapy in women with nausea and vomiting of pregnancy. Human reproduction (Oxford, England).

